# Integrating Ubuntu: embedding African philosophy into disaster risk reduction and management

**DOI:** 10.3389/fsoc.2025.1637051

**Published:** 2025-09-03

**Authors:** Wilfred Lunga, Mkhokheli Sithole, Charles Musarurwa, Eugene Nawanti Kombaté, Tendayi Marovah

**Affiliations:** ^1^Human Sciences Research Council (HSRC), Pretoria, South Africa; ^2^Faculty of Natural and Agricultural Sciences, Northwest University's African Centre for Disaster Studies (ACDS), Potchefstroom, South Africa; ^3^Faculty of Natural and Agricultural Sciences, University of Free State's Disaster Management Training Education Centre (DIMTEC), Bloemfontein, South Africa; ^4^Faculty of Commerce, National University of Science and Technology (NUST), Bulawayo, Zimbabwe; ^5^Midlands State University, Gweru, Zimbabwe; ^6^Faculty of Education, West African Science Service Centre on Climate Change and Adapted Land Use (WASCAL), Lome, Togo

**Keywords:** Southern Africa development community, disaster risk management, disaster risk reduction, Ubuntu, ideology, infrastructural power, state formation, socio-natural relations

## Introduction

Disasters, whether natural, socio-natural, or anthropogenic, are not merely technical challenges but deeply political and cultural phenomena shaped by historical and ideological contexts. In Southern Africa, Disaster Risk Reduction and Management (DRMM) has long been dominated by global, technocratic frameworks that sideline indigenous knowledge systems. Yet, as climate hazards intensify, the limitations of infrastructure-centric approaches become evident, demanding a shift toward culturally grounded resilience strategies. This opinion piece argues that integrating *Ubuntu*^1^, an African philosophy emphasizing communality, reciprocity, and shared humanity, into DRRM offers a transformative alternative to Western-centric models. While Ubuntu has influenced postcolonial governance and justice, its potential in disaster risk governance remains overlooked. The opinion piece challenges universalist risk paradigms and advocates for decolonized, participatory disaster governance where resilience emerges not from institutional power alone, but from the moral and social fabric of communities.

## Background

Disasters are frequently framed as “natural” occurrences, yet an expanding body of interdisciplinary scholarship has established that their root causes are largely social, political, and economic. Rather than being the direct result of physical hazards, disasters emerge through socially constructed systems of vulnerability shaped by governance failures, structural inequalities, and historical injustices (Blaikie et al., 2014; Tierney, 2014; Gaillard, 2010). Smith (2007) characterizes disasters as a product of a “social calculus” in which race, class, gender, and spatial exclusion determine exposure and outcomes. Similarly, Chimutina and Von Meding (2019) argue that the true disaster lies not in the hazard itself but in unsafe urban systems, failed policies, and planning deficits. In this sense, the use of the term “natural disaster” has been critiqued as obscuring the role of human agency and accountability (Von Meding, 2021).

These critiques are especially relevant in the African context, where disaster risk is tightly interwoven with legacies of colonialism, land dispossession, spatial inequality, and socio-economic marginalization (Holloway et al., 2013; Paudel et al., 2021). In countries across the Southern African Development Community (SADC), communities continue to experience cyclical floods, droughts, and cyclones, which are exacerbated by underinvestment in risk reduction, weak institutional coordination, and uneven development (Manyena et al., 2011; Mavhura, 2016; Mavhura et al., 2017). For example, climate-induced hazards in Mozambique, Malawi, and Zimbabwe have disproportionately affected low-income and peri-urban communities that are often excluded from early warning systems and post-disaster recovery (Nhamo et al., 2020b). As such, disasters in the region must be understood not as environmental shocks alone, but as the outcome of intersecting socio-political systems.

Despite this, dominant DRRM frameworks in many African states remain largely technocratic, modeled on global policy regimes and reliant on vertical, state-driven approaches. These systems emphasize institutional reforms, hazard monitoring, and infrastructure development, often without adequately integrating local realities, participatory knowledge, or culturally embedded risk management practices (Van Niekerk, 2015; Coppola, 2021). This top-down orientation is symptomatic of what Gaillard (2010) calls “epistemological colonialism,” the privileging of Western scientific models over indigenous and community-based knowledge systems.

One critically underutilized paradigm in this regard is Ubuntu, a relational African philosophy that emphasizes mutual care, interdependence, and communal responsibility (Mugumbate et al., 2024). Ubuntu frames resilience not merely as a technical or institutional output, but as a moral and collective obligation (Shawa, 2024). It challenges the individualistic assumptions of dominant DRRM models by centering solidarity, ethical reciprocity, and shared vulnerability. Empirical studies from South Africa and Mozambique have shown that communities grounded in Ubuntu practices often rely on mutual aid networks, intergenerational oral histories, and participatory rituals to prepare for and respond to disasters (Manyena, 2006). These practices not only enhance psychosocial recovery and community cohesion but also reinforce a sense of ethical responsibility for collective survival. Furthermore, international examples, such as Cuba's community-embedded disaster model, demonstrate that socially embedded, equity-driven DRRM systems can outperform resource-intensive, technocratic models in terms of preparedness and adaptive capacity (Suárez-Medina et al., 2021). In a similar vein, Ubuntu offers a context-specific and ideologically resonant framework that complements physical infrastructure with ideological investment, building resilience through both tangible systems and intangible values (Mann, 2012; Loftus, 2020).

Against this backdrop, this opinion piece argues that DRRM in Southern Africa must be reconceptualised not only through the lens of state capacity and technocratic efficiency but also through the ideological and cultural systems that shape how risk is understood, communicated, and governed. African states, particularly in the SADC region, should not be treated solely as infrastructural actors but as socio-natural entities formed through discursive, cultural, and political processes.

Thus, the key questions are how Ubuntu and African ideology inform DRRM practices, and what is the role of ideological power in shaping disaster risk governance in Africa and SADC member states?

## Methodological framework and analytical approach

This opinion piece adopts a qualitative, interpretive research design rooted in African relational epistemologies and critical disaster studies to interrogate how disaster experiences in Southern Africa are culturally mediated. Grounded in the interpretivist view that reality is socially constructed and shaped by lived experience (Lotz-Sisitka et al., 2013), the study frames Ubuntu not only as a normative philosophy but as an evolving social practice that informs communal resilience. Interpretations of Ubuntu as told through oral narratives, communal rituals, and political discourse reveal how communities negotiate meaning in the face of crisis (Mugumbate and Nyanguru, 2013; Mugumbate and Chereni, 2020; Ndlovu-Gatsheni, 2018). Integrating over 30 scholarly sources, including peer-reviewed literature, SADC policy documents, and African philosophical texts, the analysis builds a textured understanding of locally grounded disaster governance.

This approach is further informed by the authors' diverse professional backgrounds in education and disaster risk management, enabling a cross-sectoral perspective on the issues under discussion. Critical Discourse Analysis (Fairclough, 2013) was applied to state and institutional texts, while thematic analysis (Kupika et al., 2019) identified patterns in community-based narratives and indigenous knowledge systems. The piece also draws on relational ethics (McCubbin and Moniz, 2014), privileging reciprocity, contextual sensitivity, and epistemic justice. In doing so, it aligns with calls for research that braids Ubuntu with Indigenous knowledge to more effectively interpret DRRM issues (Huambachano, 2018; Scarano, 2024; Intergovernmental Panel on Climate Change, 2022). Ultimately, this approach challenges dominant Eurocentric disaster governance models by reframing resilience as an ethical, collective practice rooted in African ways of knowing and being (Darder, 2018; Ndlovu-Gatsheni, 2018).

## Unpacking and operationalising Ubuntu in DRMM in Africa and the SADC region

Ubuntu, a foundational African socio-cultural philosophy prevalent among Bantu-speaking communities, offers a relational and ethical paradigm for disaster risk management (DRM) and disaster risk reduction (DRR) across the continent. Rooted in the maxim “*Umuntu ngumuntu ngabantu*” (“A person is a person through others”), Ubuntu emphasizes interconnectedness, collective agency, and mutual care (Tutu, 2009; Shadrach, 2025; Ajitoni, 2024; Mugumbate et al., 2024). It frames individuals not as isolated agents but as integral members of social networks, where communal well-being defines resilience. This ontological orientation challenges dominant technocratic and top-down DRM models by privileging participatory governance, especially involving historically marginalized actors such as women, elders, and youth (Nhemachena and Mlambo, 2020). Empirical evidence from Southern and Eastern Africa confirms Ubuntu's operational value. In Malawi, Kenya, and the Sahel, grassroots actors, including traditional leaders and local elders, co-produce risk knowledge, establish hybrid early warning systems, and conduct participatory vulnerability mapping, demonstrating how Ubuntu principles enhance legitimacy, inclusivity, and contextual relevance (Okoli, 2020; Saber et al., 2025). These practices exemplify a decolonial shift in disaster governance, rooted in Africa's long-standing traditions of collective problem-solving.

Ubuntu-informed DRRM also rectifies structural asymmetries by legitimizing indigenous institutions such as village councils, traditional courts, and community forums as essential governance actors (Teleki and Kamga, 2020). This model embeds equity and cohesion into routine risk governance by amplifying local voices and reinforcing the value of indigenous authority systems (Mawere and Mukonza, 2024; Mangena, 2019). Case studies from Ethiopia's drought adaptation and Mozambique's post-cyclone recovery affirm the efficacy of Ubuntu-based approaches in advancing self-determined, justice-oriented resilience (Asante, 2007a,b). Rather than rejecting science, Ubuntu facilitates a synthesis between indigenous knowledge and modern technologies. Hybrid approaches, such as Zimbabwean farmers combining traditional rain signs with satellite forecasts or South African communities developing evacuation protocols with risk scientists, illustrate this convergence and the resulting increase in community trust and uptake of DRR measures (Motsumi and Nemakonde, 2024; Muhame et al., 2024).

As climate change escalates the frequency and intensity of extreme weather events, particularly in the SADC region, Ubuntu presents a compelling alternative to externally imposed resilience frameworks. Reframing resilience as a socially embedded, ethically grounded, and communally owned process, Ubuntu enables the transition from reactive to proactive, culturally attuned disaster governance. Its widespread operationalisation could serve as a cornerstone for decolonised, people-centered climate adaptation strategies in Africa (Ndlovu-Gatsheni, 2018; Newman et al., 2023). This turn toward endogenous approaches is not without precedent. Historical experiences across the region, particularly during the post-independence period, demonstrate that resilience in Southern Africa has long been underpinned by ideologically driven, socially embedded practices.

## Infrastructural and ideological expansion (1970s−1990s)

Between the 1970s and 1990s, Southern African states such as Mozambique, Malawi, Zimbabwe, and South Africa expanded their infrastructural power through the construction of roads, irrigation schemes, flood control systems, and early warning networks. These developments enhanced state capacity to manage environmental risks and extended administrative presence into rural areas (Mann, 2012; Stevens and van Koppen, 2015). While infrastructure helped stabilize post-colonial governance, it did not by itself confer legitimacy. What emerged alongside this expansion was a parallel investment in ideological power, the ability of the state to shape meaning, identity, and ethical responsibility about disasters. During this period, communities across the region engaged in rich cultural production to make sense of ecological disruption. Oral storytelling, commemorative rituals, and symbolic acts were central to framing disasters not simply as natural events but as historical and moral experiences. In Zimbabwe, flood narratives and other hazards were tied to the liberation struggle, positioning environmental crises as metaphors for colonial injustice and state neglect (Mavhura et al., 2015a,b). In Mozambique, disaster education was historically rooted in socialist-era cultural campaigns that fostered resilience through collective identity, solidarity, and national consciousness (Müller, 2014). Similar trends across the SADC region reveal that community-based DRR was deeply embedded in post-liberation political discourse, reinforcing social cohesion as a core resilience mechanism rather than relying solely on infrastructure (Artur and Hilhorst, 2012). This underscores how DRR strategies in Southern Africa were shaped as much by socio-political identity as by technical preparedness.

Such ideological framing was not unique to these countries. The 1984 Ethiopian famine became a symbolic moment for national solidarity and citizenship, while in Malawi, community flood rituals combined spiritual interpretation with a critique of state failure (Steinforth, 2021). These cases demonstrate that disasters are both material and symbolic, shaping how people engage with the state and with each other. Although disaster DRRM across Africa remains largely state-led, often privileging centralized, technocratic approaches (Cabane, 2023), this perspective overlooks the significance of ideological and cultural governance. Mann's (2012) distinction between authoritarian and infrastructural power offers useful insights into state capacity, but more recent scholarship emphasizes the relational and contested nature of statehood, where infrastructure is also a site of meaning-making (Meehan, 2014a,b; Fortin-Rittberger, 2014). Elden (2019) and Cederlöf (2023) argue for understanding territory as shaped by sociotechnical and ecological relations, aligning with critical disaster studies that reject depoliticised, technocratic models (Oliver-Smith, 2004, 2013; Bankoff et al., 2013).

In post-colonial African contexts, disasters are inseparable from histories of marginalization and uneven development. In Zimbabwe, for instance, land dispossession has heightened flood vulnerability (Mavhura et al., 2015a,b), while in Kenya, drought exposure is exacerbated by governance failures (Mutua, 2016; Walshe et al., 2022a,b,c). In this context, ideological narratives are crucial in shaping how citizens interpret state legitimacy and their ethical roles in disaster response. Ultimately, disaster governance in Africa is best understood as a co-constitution of infrastructural and ideological power. Infrastructure enables logistical intervention, but ideology gives meaning to disaster and resilience. It is through this interplay between roads and rituals, policy and storytelling, that states and communities co-produce disaster governance. Recognizing this dynamic is essential for building inclusive, context-sensitive DRR strategies that reflect the lived realities and philosophical foundations of African societies.

## Decision-making and state territoriality in African DRRM

Understanding disaster governance in Africa as both infrastructural and ideological compels us to interrogate how authority is exercised across space. State responses are shaped not only by hazard exposure but by how territorial presence is asserted, who is governed, where, and through what mechanisms. These spatial dynamics often reproduce uneven geographies of power, with peripheral regions facing limited investment, militarized responses, or delayed intervention. Territoriality thus becomes a critical lens through which to assess whose risks are prioritized and whose knowledge is legitimized. Crucially, effective DRRM hinges on bridging centralized authority with decentralized, participatory mechanisms.

Legal frameworks such as South Africa's Ndapassoa (2023) institutionalize multi-level governance, assigning responsibilities across national, provincial, and local tiers (South African Government, 2002). These statutes embed accountability while reflecting Ubuntu's ethos of mutual responsibility. In Kenya, Uganda, and Malawi, local DRR committees collaborate with traditional leaders and NGOs to localize risk planning (Fortnam et al., 2020). Mozambique's DRM committees co-produce evacuation protocols with meteorological agencies, exemplifying hybrid governance models that blend local and scientific knowledge.

Education reinforces this model. DRRM is now embedded in national school curricula across Kenya, Ethiopia, and South Africa, cultivating risk literacy among youth. Informal systems, rituals, storytelling, and intergenerational knowledge further consolidate cultural resilience (Mutasa et al., 2022a; Ncube-Phiri et al., 2015). Together, these layers reveal that Africa's disaster governance is not merely technocratic; it is relational, ethical, and deeply grounded in lived realities.

## Reframing DRRM within the spirit of Ubuntu

This relational paradigm finds powerful expression in Ubuntu, a philosophical and ethical foundation centered on interdependence, care, and collective responsibility. In the African context, disasters are not just environmental disruptions but profoundly moral events, interpreted and managed through communal worldviews. Ubuntu offers more than a normative vision it constitutes a governing logic for DRRM, reframing resilience as a shared obligation rather than a technical outcome (Ndlovu-Gatsheni, 2018; Newman et al., 2023).

Across Africa, DRR strategies increasingly integrate Indigenous Knowledge Systems (IKS) with scientific innovations. Practices like foggaras in North Africa, *zai pits*^2^ in West Africa, and intercropping across the continent exemplify longstanding adaptive expertise (Reij et al., 1996; Mapfumo et al., 2016). These are now enhanced by geospatial forecasting, drought-resistant seeds, and satellite early warning systems (Nyong et al., 2007; Hiwasaki et al., 2014a,b). This hybridization fosters resilient systems attuned to both environmental shifts and cultural logics (Mercer et al., 2010).

The operational strength of Ubuntu was evident during Cyclone Idai in 2019, when grassroots rescue and reconstruction in Mozambique, Zimbabwe, and Malawi mobilized communal labor and social solidarity. In Zimbabwe's Chimanimani district, revived protection mechanisms drew on kinship and local leadership (Munsaka et al., 2021; Marango and Chitongo, 2021; Dube et al., 2021). In Mozambique, *mutirão*^3^ traditional work groups led clean-up and rebuilding without external aid. South Africa's *ukusisa*^4^ livestock-sharing practice and Botswana's mephato labor formations further demonstrate how Ubuntu enables localized resilience (Ketlhoilwe and Jeremiah, 2016). In Zambia's Zambezi River Basin, kinship-based early warning systems illustrate how relational governance ensures timely and inclusive risk communication (Collins et al., 2024).

These practices highlight how Ubuntu cultivates ethical subjectivities that extend beyond technical compliance, fostering disaster responses rooted in relational obligation and social cohesion. The concept of the resilient citizen thus shifts from an individualized, policy-driven construct to one grounded in communal ethics and shared agency. Through storytelling, oral histories, and ritual, communities preserve and transmit generational knowledge on droughts, floods, and coping strategies, reinforcing both preparedness and cultural continuity (Mugumbate and Nyanguru, 2013; Mugumbate et al., 2024). Communal rituals play a dual psychosocial and ecological role. Ceremonies performed at the onset or conclusion of a disaster offer symbolic restoration and emotional healing, honor ancestral wisdom, and often embed actionable preparedness strategies. These rituals operate as cultural infrastructures of resilience, maintaining a moral ecology of care and reciprocity (Trosper, 2009; von Heland, 2011).

Governments have also leveraged Ubuntu in political discourse and disaster campaigns to enhance public cooperation. South Africa's Working for Water programme linked ecological restoration with job creation and ethical land stewardship, offering a model of participatory resilience aligned with Ubuntu (Khomba, 2024). Similar messaging is reflected in disaster education programmes in Mozambique and Malawi, which use local proverbs and communal narratives to frame preparedness as a civic and moral responsibility. There is a growing scholarly consensus that social cohesion, trust, and indigenous knowledge, all central to Ubuntu, are critical enablers of effective DRM. Sobhaninia (2024), emphasizes how cohesion accelerates post-disaster recovery and advocates for the formal integration of indigenous knowledge into DRR policy. Evidence also shows that Ubuntu-based, community-led systems reduce dependency on external aid and foster long-term transformation (Manyena et al., 2011). Embedding Ubuntu principles into formal Disaster Risk Management (DRM) frameworks presents a transformative opportunity to decolonize disaster resilience in Southern Africa. This approach shifts communities from passive aid recipients to active ethical agents, leveraging indigenous knowledge systems and collective social structures. As shown in Table 1, the integration of Ubuntu into DRM governance aligns state structures, policy frameworks, and local practices while affirming African philosophical foundations.

**Table 1:** Ubuntu in DRM governance - key dimensions.

**Governance level**	**Structural mechanism**	**Ideological basis**	**Ubuntu presence**
National	Legal frameworks (e.g., Disaster Management Acts)	Communal responsibility, shared humanity	Policy language emphasizing collective duty
Local/Decentralized	District disaster committees, community-based DRR	Participatory decision-making, reciprocity	Grassroots risk assessments, mutual aid systems
Cultural/Educational	DRR school curricula, public awareness campaigns	Interconnectedness, moral obligation	Indigenous storytelling, disaster memorial rituals

The data presented in Table 1 illustrates how integrating Ubuntu into disaster governance bridges formal institutional structures with culturally embedded forms of resilience, offering a decolonial alternative to dominant, technocratic DRM models. At the national level, legislative frameworks such as South Africa's Disaster Management Act and Mozambique's Disaster Risk Management Act explicitly articulate communal responsibility, mirroring Ubuntu's ethic of shared humanity and mutual obligation. This represents a shift in governance logic: state mandates are reinterpreted not as bureaucratic imperatives but as collective moral duties rooted in African relational ontologies.

Decentralized governance further demonstrates Ubuntu's operational strength. Local disaster committees such as Malawi's Civil Protection Units apply participatory and reciprocal decision-making processes that empower communities to co-produce risk management strategies. Unlike conventional top-down systems, these structures frame resilience as a social contract, grounded in mutual aid rather than external intervention. Similarly, the integration of cultural and educational elements, such as Kenya's DRR curriculum infused with storytelling and ritual, reinforces intergenerational memory and Ubuntu's principle of interconnectedness.

This analysis underscores Ubuntu's dual function: as a normative compass, shaping ethical values of care, dignity, and solidarity, and as a practical toolkit, enabling grassroots resilience through culturally attuned action. For SADC states, embedding Ubuntu into DRM is not merely a gesture toward cultural preservation; it represents an epistemic reclaiming of sovereignty in disaster governance. Institutionalizing Ubuntu principles such as reciprocity, collective dignity, and interdependence reorients DRM systems toward inclusive, participatory, and locally grounded forms of governance.

Table 2 further elaborates this analysis by mapping disaster governance frameworks across SADC countries, highlighting varying degrees of integration of indigenous philosophies like Ubuntu within national and subnational DRM architectures. This comparative perspective affirms that culturally embedded systems are not peripheral but foundational to building equitable and sustainable resilience in the region.

**Table 2:** Governance structure, ideology, and presence of Ubuntu.

**Country**	**Governance structure**	**Ideology**	**Ubuntu presence**	**Sources**
**South Africa**	Decentralized disaster governance under the Disaster Management Act (2002); responsibilities shared across national, provincial, and municipal spheres	Democratic constitutionalism, developmental state with strong community participation	**Yes** - Ubuntu philosophy is explicitly referenced in community disaster frameworks and social cohesion policies	South African Government, 2002; Van Niekerk, 2015; Holloway, 2003
**Mozambique**	Centralized under INGD with provincial and district implementation; strong recovery structures post-disaster (e.g., after cyclones)	Post-conflict socialist roots evolving toward participatory governance in DRR	**Partially**, traditional communal values influence local responses, but not formally codified as Ubuntu	Artur and Hilhorst, 2012
**Malawi**	Department of Disaster Management Affairs (DoDMA); local structures through District Civil Protection Committees	Multiparty democracy with decentralization and community DRR	**Yes** - Community-based disaster management is rooted in African communalism traditions akin to Ubuntu	Pangapanga-Phiri et al., 2022; Kayamba-Phiri et al., 2023; Dewa et al., 2021
**Zimbabwe**	Centralized Civil Protection Unit (CPU) with provincial and district committees	Post-liberation ideology with centralized control; mixed with grassroots participation	**Partially** - Spirit of communalism strong at local level; Ubuntu values inform local disaster recovery efforts	Chikoto and Sadiq, 2012; Gwimbi, 2007
**Tanzania**	Prime Minister's Office Disaster Management Department (PMO-DMD) coordinates DRR; decentralized to villages	Democratic socialism heritage; emphasizes grassroots resilience	**Yes** -Ujamaa philosophy (similar to Ubuntu) underpins community participation in DRR	West et al., 2018; Daly et al., 2016
**Botswana**	National Disaster Management Office (NDMO) coordinated under the Office of the President, district-led response structures	Liberal democracy with strong national coordination	**Partially** -Traditional leadership structures promote communal values similar to Ubuntu, but not formally integrated	Ugwu et al., 2022; Hammood et al., 2025
**Madagascar**	Bureau National de Gestion des Risques et des Catastrophes (BNGRC) leads coordination; village risk committees are active	Fragile democracy with international humanitarian partnerships	**Limited** -Communal approaches exist informally; Ubuntu is not a formalized part of governance	November and Leanza, 2014; Urban and Ratsimanetrimanana, 2015.

Disaster governance in Southern Africa reflects diverse institutional architectures and varying degrees of integration of indigenous philosophies such as Ubuntu. In South Africa, the Disaster Management Act (2002) mandates a decentralized, multilevel governance structure spanning national, provincial, and municipal levels. Rooted in a democratic constitutional framework and developmental state model, this structure emphasizes participatory governance. Ubuntu is formally embedded in community disaster frameworks, reinforcing social cohesion and collective resilience (South African Government, 2002; Van Niekerk, 2015; Holloway, 2003). In contrast, Mozambique maintains a centralized model under the National Institute for Disaster Management (INGD), with implementation cascading to provincial and district levels. While disaster recovery mechanisms are well-established, particularly in cyclone-prone zones, the influence of post-socialist ideology and traditional communal practices persists. However, Ubuntu is not formally institutionalized in policy (Artur and Hilhorst, 2012; Marango and Chitongo, 2021).

Malawi employs a hybrid model where the Department of Disaster Management Affairs (DoDMA) collaborates with decentralized District Civil Protection Committees. Its multiparty democracy enables bottom-up DRR strategies informed by communal traditions akin to Ubuntu, enhancing local ownership and shared responsibility (Dewa et al., 2021; Chanza and de Wit, 2016a,b). Zimbabwe's disaster governance remains centralized under the Civil Protection Unit (CPU), supported by provincial and district structures. The system reflects post-liberation governance dynamics, combining state-centric authority with grassroots participation. Ubuntu values are deeply embedded in community responses, enhancing social solidarity during crises (Ajitoni, 2024; Gwimbi, 2007; Dube et al., 2021).

In Tanzania, the Prime Minister's Office Disaster Management Department (PMO-DMD) oversees DRR, with responsibilities devolved to village-level actors. Influenced by Ujamaa a philosophy aligned with Ubuntu, Tanzania's disaster governance integrates democratic socialist ideals, promoting collective resilience through community-based approaches (Mlingwa, 2024; Kamanyi, 2024). Botswana, operating within a liberal democratic framework, includes traditional leadership in DRM processes. While communal values akin to Ubuntu are present, they are not formally embedded within national DRM policy (Dennison and Coldwell, 2025). Similarly, Madagascar's disaster governance, coordinated by the Bureau National de Gestion des Risques et des Catastrophes (BNGRC), relies heavily on village-level committees and international support. Though communal practices persist, Ubuntu or equivalent indigenous philosophies remain informal and largely absent from official policy frameworks.

## Findings and observations

This opinion piece identifies four interrelated themes that demonstrate how disaster risk governance in the SADC region is mediated not only by infrastructure and policy but also by cultural ethics, indigenous knowledge, and ideological frameworks, particularly those informed by Ubuntu. The findings reflect a shift from technocratic risk management to resilience grounded in communal identity, ethical subjectivity, and locally embedded epistemologies.

## Resilience as cultural and ethical identity

Across Southern Africa, resilience is not only a functional response to hazards but also a culturally constructed identity rooted in Ubuntu, an ethic that foregrounds interdependence, solidarity, and moral obligation. Community-based disaster responses in Malawi, Zimbabwe, and Mozambique following Cyclone Idai illustrate this principle: mutual aid networks, communal rebuilding, and oral knowledge-sharing practices emerged organically in the absence of formal aid (Munsaka et al., 2021; Dube et al., 2021). In Botswana and South Africa, traditional systems like *mephato*^5^ (age-based labor), and *ukusisa* (cattle sharing) provides models of embedded resilience that blend ecological stewardship with social reciprocity (Morton, 2012). These findings align with the concept of “culturally embedded resilience” (Bankoff, 2019), wherein community members do not merely adapt to risk but embody resilience as a lived ethical practice passed through generations. Ubuntu thus positions resilience not as an individual trait but as a collective moral identity that binds communities in shared responsibility (Tutu, 2009).

## Disasters as ideologically mediated events

Disasters in the region are not merely physical occurrences; they are ideologically framed within broader narratives of state legitimacy, environmental justice, and historical memory. During the 1980s and 1990s, many Southern African states expanded infrastructural power through flood control and warning systems. Yet, this material expansion was paralleled by symbolic cultural production, including flood myths, liberation-era storytelling, and rituals that linked environmental shocks to political critique and spiritual imbalance (Mavhura et al., 2015a,b; Mboweni and De Crom, 2016). In Mozambique and Ethiopia, disasters were used to construct narratives of nationhood, state responsibility, and communal sacrifice (Müller, 2014). These ideologically charged representations mediate how citizens understand risk, interpret state actions, and enact responses, thus making disaster governance a site of moral negotiation as much as technical execution (Loftus, 2015; Oliver-Smith, 2015).

## DRRM as a moral and political space

Ubuntu-based DRM frameworks highlight that disaster governance in Africa operates as a moral economy, not simply a bureaucratic or technocratic function. Participatory risk assessments in Kenya, Malawi, and Uganda integrate elders, youth, and women, reflecting Ubuntu's emphasis on collective wisdom and ethical inclusion (Hiwasaki et al., 2014a,b; Mwangi et al., 2022, 2021). Post-disaster recovery in Zambia and Rwanda illustrates how communal solidarity and shared healing displace hierarchical aid models with endogenous, dignity-based recovery processes (Habimana and Biracyaza, 2023; Gregory, 2013).

DRR education models also reflect this shift: Kenya and South Africa have introduced curricula that blend hazard science with oral storytelling, ancestral rituals, and proverbs reaffirming that preparedness is a culturally transmissible moral obligation (Mutasa et al., 2022b; Leal Filho et al., 2022b,a). These findings support the view that resilience must be seen as a negotiated moral practice, co-produced by community actors and institutions (Nhamo et al., 2020b; Mugumbate and Nyanguru, 2013).

## Contrasts with technocratic models

State-centric, technocratic DRM approaches often marginalize or underutilize indigenous epistemologies, leading to legitimacy gaps and missed opportunities for deeper civic engagement. While centralized policies like South Africa's Ndapassoa (2023) offer structural coherence, their effectiveness is enhanced only when harmonized with local participatory structures such as ward-level councils and traditional authorities (Sipondo, 2025; Nhamo et al., 2020a). Conventional models typically treat vulnerability as a technical deficit rather than a relational condition shaped by exclusion, culture, and memory (Chikoto and Sadiq, 2012). In contrast, Ubuntu-informed systems such as Malawi's civil protection committees or Mozambique's hybrid early warning systems highlight how trust, relational ethics, and cultural legitimacy are indispensable to long-term resilience (Chanza and de Wit, 2016a,b; Leal Filho et al., 2022b,a).

Hybrid DRM models that combine satellite data with indigenous indicators such as animal migration or local calendar systems in Zimbabwe and Tanzania demonstrate Ubuntu's capacity to translate risk into action through culturally resonant pathways (Ndlovu-Gatsheni, 2020). These examples show that resilience is not simply built; it is socially and ethically negotiated. Table 3 shows how disaster risk governance in the SADC region is mediated not only by infrastructure and policy but also by cultural ethics, indigenous knowledge, and ideological frameworks, particularly those informed by Ubuntu.

**Table 3:** Ubuntu in DRRM practices.

**Theme**	**Key principles**	**Examples**
Participatory community engagement in DRM	Community mapping, risk profiling, and hazard monitoring can be participatory, leveraging local knowledge and strengthening communal ties	In Malawi's flood-prone districts, villagers collaborate with NGOs to map flood zones and share early warnings using traditional signals and mobile alerts
Embedding Indigenous Knowledge Systems (IKS) into DRM/DRR policies	Ubuntu promotes respect for heritage and local wisdom	Mozambique's communities blend traditional cyclone warning signs with meteorological forecasts for earlier preparation
Ubuntu as an ethical framework for DRM/DRR governance	Encourages ethical leadership based on solidarity and collective wellbeing	In South Africa, Ubuntu-based interventions could transform informal settlement risk strategies through neighborhood committees and collective evacuation planning
Building resilience through Ubuntu-driven social capital	Ubuntu strengthens social capital, critical for disaster resilience	After Cyclone Idai (2019), informal Ubuntu networks delivered aid faster in remote Mozambique/Zimbabwe communities than formal agencies
Ubuntu-driven risk communication and public education	Uses storytelling, rituals, and collective memory to make warnings culturally resonant, enhancing community awareness and preparedness	In rural Zimbabwe, elders use proverbs and community gatherings to communicate drought risks and adaptive practices, increasing local preparedness and response

The table shows that participatory DRM leverages local knowledge (e.g., Malawi's community flood mapping) while integrating Indigenous wisdom (e.g., Mozambique's hybrid cyclone warnings). Ubuntu ethics strengthen governance (e.g., South Africa's collective evacuation plans) and social resilience (e.g., post-Cyclone Idai community aid networks). Culturally rooted communication (e.g., Zimbabwe's drought proverbs, i.e, “*Usatuke chidembo une vakwegura mumba*^6^ (“Do not insult a skunk if you have elders in your home”) enhances preparedness. Merging science, local knowledge, and Ubuntu fosters inclusive, adaptive disaster resilience.

## Disasters as revolutionary struggles and the ethical-political dimensions of Ubuntu

The findings of this opinion piece reveal that DRRM in Southern Africa is more than operational fields; they are ideological terrains where competing paradigms of sovereignty, knowledge, and community are negotiated. At the heart of these negotiations lies Ubuntu: a decolonial ethic of interdependence that transforms disaster response from a technocratic exercise into a revolutionary act of collective survival. From a historical and political perspective, disasters in Africa have long been framed not merely as ecological crises but as ideological inflection points. The Sahelian droughts of the 1970s and the Mozambique floods of 2000 catalyzed post-independence political realignments and stimulated regional integration, transforming natural hazards into moments of state consolidation and Pan-African solidarity (Neves, 2024). These events echoed the revolutionary environmentalism articulated by leaders like Nyerere and Sankara, who viewed ecological mastery as integral to sovereignty and justice (Sankara, 2007; Nkrumah, 1963; Nyerere, 1968). Their philosophies positioned resilience not as adaptation to environmental variability, but as a conscious, collective struggle for liberation.

Ubuntu, in this revolutionary register, becomes more than a cultural idiom; it is an ideological counterweight to colonial technocratic governance. It reframes the “resilient citizen” as an ethical subject bound by duty, solidarity, and shared responsibility (Mugumbate and Nyanguru, 2013; Ndlovu-Gatsheni, 2018). Ubuntu-based disaster responses, such as those observed in Chimanimani or the Zambezi River Basin, exemplify a form of grassroots governance where resilience emerges through communal labor, mutual aid, and culturally embedded risk knowledge (Munsaka et al., 2021; Masunda and Chawhanda, 2025).

However, the translation of Ubuntu ideals into practical DRRM governance is far from seamless. One critical tension lies in the epistemological divide between Indigenous Knowledge Systems (IKS) and modern scientific paradigms. While Africa's rich repertoire of traditional ecological practices, such as agroecological intercropping, rain rituals, and oral early warning systems, has proven resilient over generations (Mapfumo et al., 2016; Reij et al., 1996), technocratic systems continue to dominate disaster governance. Satellite-based models and national command structures often marginalize local knowledge holders, perpetuating colonial hierarchies of expertise and authority (Mercer et al., 2010; Leach et al., 1999).

This epistemic tension is emblematic of a broader structural contradiction: Ubuntu emphasizes relationality and inclusion, yet disaster governance in much of the SADC region remains centralized, donor-driven, and vulnerable to elite capture (Okunola, 2025; Sipondo, 2025). Community-based disaster committees, while rhetorically inclusive, often lack fiscal autonomy and legal mandate. Moreover, the political economy of DRR framed within neoliberal climate finance regimes rarely accommodates Ubuntu's long-term, intergenerational ethics (Odora Hoppers, 2002; Bankoff, 2001). Despite these challenges, hybrid models are emerging. In Zimbabwe and South Africa, farmers have combined traditional forecasting with satellite data, while community risk mapping initiatives in Kenya and Uganda co-produce knowledge between elders, youth, and scientists (Mwangi et al., 2022, 2021; Ndlovu-Gatsheni, 2020). These cases represent not just technical innovations but epistemological reconciliation efforts to democratize resilience by grounding it in local meaning-making and historical consciousness.

Moreover, the ideological framing of disasters as revolutionary struggles remains deeply salient. Whether through the symbolism of the Great Green Wall, the politicization of droughts as state failure, or the memorialization of flood victims in collective rituals, African societies continue to interpret environmental crises through the lens of sovereignty, justice, and self-determination (Nyong et al., 2007; Maathai, 2003). Ubuntu, in this context, functions both as a moral compass and a mobilizing discourse that binds ecological ethics to political emancipation. Nevertheless, for Ubuntu to move beyond symbolic invocation, it must be institutionally operationalized. This requires dismantling the epistemic hierarchies that sideline local knowledge, redistributing resources to empower grassroots institutions, and reorienting disaster policy toward long-term relational care rather than short-term technical fixes. As Ndlovu-Gatsheni (2020) argues, decolonial resilience demands a rupture with the logics of coloniality that frame African communities as passive recipients of aid rather than as epistemic agents.

In sum, the integration of Ubuntu into DRRM is both a political necessity and a philosophical imperative. It offers a pathway toward epistemic justice, where Africa's historical struggles against colonial subjugation are mirrored in contemporary struggles against environmental precarity. Disasters, framed through Ubuntu, are not merely threats; they are revolutionary opportunities to reimagine governance, reclaim cultural agency, and build systems of resilience that are ethically grounded, locally owned, and globally just.

## Conclusion

This opinion piece has critically examined how Ubuntu and broader African ideological frameworks fundamentally inform DRRM practices within Africa, particularly in the SADC region. The piece aimed at answering the two key guiding questions, which were (a) how does Ubuntu as an African ideology inform DRRM practices, and (b) what is the role of ideological power in shaping disaster risk governance in Africa and SADC states? It has been demonstrated that disaster governance in African contexts transcends technocratic and material dimensions to become a deeply ideological and moral terrain, where collective identity, cultural resilience, and historical struggles for sovereignty shape responses to floods, droughts, and other hazards. Ubuntu, with its core tenets of interconnectedness, mutual aid, and shared responsibility, provides a culturally embedded normative and practical foundation that fosters participatory, community-led resilience. Crucially, the role of ideological power in shaping disaster risk governance manifests through the co-production of knowledge, legitimization of indigenous institutions, and the reframing of disasters as collective ethical challenges rather than mere technical problems. This epistemic and political recalibration challenges conventional state-centric, technocratic DRM models that often overlook socio-cultural complexities and opportunities for deeper public engagement. Embedding Ubuntu thus emerges not only as a decolonial imperative but as a strategic pathway to developing contextually relevant, equitable, and sustainable disaster governance systems in Africa.

## Recommendations

To effectively operationalize Ubuntu within Disaster Risk Management (DRM) and Disaster Risk Reduction (DRR) frameworks across Africa and the SADC region, a multidimensional and culturally grounded approach is required. Firstly, governments should integrate Ubuntu's core values—solidarity, mutual aid, and collective dignity into national DRM policies and legislation. This calls for inclusive dialogue platforms that bring together policymakers, traditional leaders, civil society, and academics to ensure that disaster governance reflects the lived realities and socio-cultural norms of African communities (Chanza and de Wit, 2016a,b; South African Government, 2002). Equally important is the revitalization and hybridization of Indigenous Knowledge Systems (IKS) with scientific innovation. Documenting traditional environmental practices and co-developing hybrid approaches that combine indigenous wisdom with tools such as satellite-based early warning systems and climate models, communities can enhance the legitimacy and usability of DRR strategies. Collaborative partnerships between elders, local actors, and scientists are critical in this process (Mwangi et al., 2022, 2021; Leal Filho et al., 2022b,a).

Strengthening the capacity of community-based DRM structures is also essential. Local disaster committees and civil protection units must be resourced, trained, and legally empowered to design and lead tailored preparedness and response activities. This localized governance aligns with Ubuntu's participatory ethos, reinforcing grassroots ownership of resilience efforts (Sipondo, 2025). Education and public awareness campaigns should further embed Ubuntu ethics in both formal and informal systems. Schools, community centers, and media platforms must incorporate storytelling, intergenerational dialogue, and culturally resonant narratives that foster collective responsibility and environmental stewardship. This moral framing of resilience builds ethical subjectivities necessary for long-term adaptation (Bankoff, 2019; Mugumbate and Nyanguru, 2013).

In light of the transboundary nature of climate-related disasters, Ubuntu's emphasis on interconnectedness offers a powerful framework for regional cooperation. Strengthened cross-border coordination through SADC and AU initiatives can facilitate joint risk assessments, resource pooling, and community-led transnational responses, reinforcing continental solidarity (African Union, 2014; Chirwa et al., 2023). Finally, ensuring sustained implementation requires mobilizing equitable and Ubuntu-informed financing. This entails reimagining disaster funding models that prioritize vulnerable populations, support endogenous innovation, and promote justice-centered approaches. By aligning political will and financial mechanisms with Ubuntu values, African states can move beyond externally imposed, technocratic responses and build locally embedded systems of resilience (Adeola, 2024; Ndlovu-Gatsheni, 2018).

Together, these measures offer a pathway toward decolonised, inclusive, and ethically grounded disaster governance that resonates with Africa's philosophical and cultural heritage.

## Possible areas of further research

Further research should focus on translating Ubuntu principles as mutual aid and collective responsibility, into actionable DRR policies rooted in local realities. Studies are needed to explore how indigenous knowledge can be effectively combined with modern technologies like GIS and early warning systems to build hybrid, culturally grounded resilience strategies. Comparative analyses of community-led vs. state-led responses can shed light on which models foster stronger long-term recovery, particularly in rural contexts where Ubuntu values thrive. Gender-focused research is essential to highlight the leadership roles of women and marginalized groups in Ubuntu-based DRR. Ubuntu also offers a compelling lens for advancing climate justice and informing equitable, reparative global policies. In addition, examining cross-border cooperation and sustainable, community-centered financing models will be key to embedding Ubuntu more deeply into regional disaster governance.

## Footnotes

^1^*Ubuntu* in deep African thinking is a profound life philosophy, ethical compass, social practice, and political framework that emphasizes that our humanity is inextricably tied to the humanity of others. It is a living knowledge system that continues to shape African responses to governance, conflict, education, disaster resilience, and collective well-being. At its core, Ubuntu is best captured by the isiZulu/Xhosa phrase: “*Umuntu ngumuntu ngabantu*” - “A person is a person through other persons.”

^2^*Zai pits* are traditional planting basins widely used in arid and semi-arid regions of Africa, particularly in Burkina Faso, Niger, and parts of Zimbabwe, to rehabilitate degraded land and improve agricultural productivity. Typically measuring 20–40 cm wide and 10–20 cm deep, these pits are dug during the dry season and filled with organic matter such as manure or compost. When the rains arrive, the pits capture and retain water around the root zones of crops like sorghum, millet, and maize, enhancing moisture availability and nutrient concentration.

^3^*Mutirão* is a Portuguese term used in Mozambique to describe a form of voluntary, communal labor mobilized for public purposes—often in the aftermath of disasters. This practice involves neighbors collaborating to clear debris, rebuild homes, or repair infrastructure without formal compensation. In disaster studies, mutirões have been recognized as grassroots recovery mechanisms driven by Ubuntu principles.

^4^*Ukusisa* is an IsiXhosa livestock-sharing practice prevalent in South Africa's Eastern Cape. During periods of hardship such as drought, pastoral households share cattle—redistributing herds to those whose animals are unhealthy or weakened, thereby preserving livelihoods through communal risk-pooling. This form of mutual aid enhances resilience by leveraging social bonds, traditional norms, and shared responsibility.

^5^*Mephato* refers to traditional age-based regiments or groups in Southern African societies, particularly among the Tswana people of Botswana, Lesotho, and parts of South Africa. While the term is not widely used across all of South Africa, it is culturally significant in Basotho and Batswana traditions, which extend into the North West Province, Free State, and Limpopo in South Africa.

^6^“*Usatuke chidembo une vakwegura Mumba* “cautions against disregarding traditional ecological wisdom, symbolically referring to how elders, through their experience, understand seasonal cycles and drought signs underscoring the epistemic authority of indigenous elders in DRRM.
